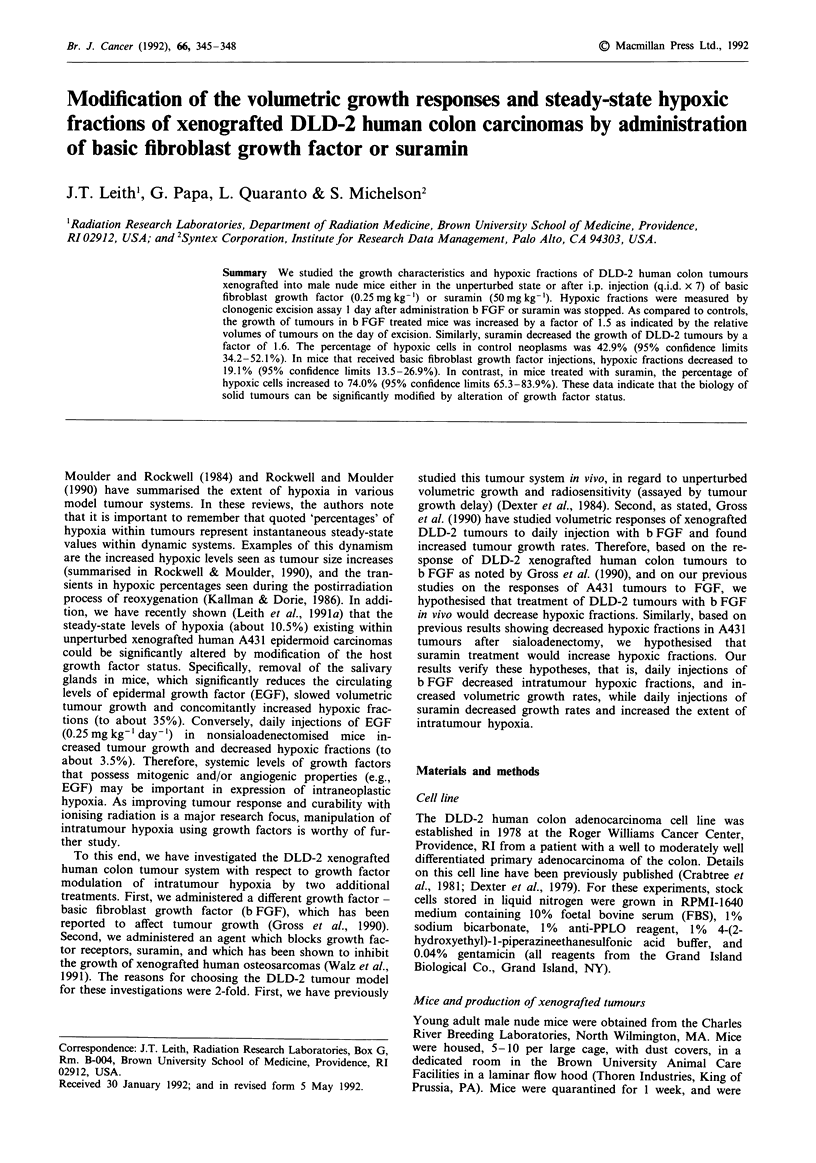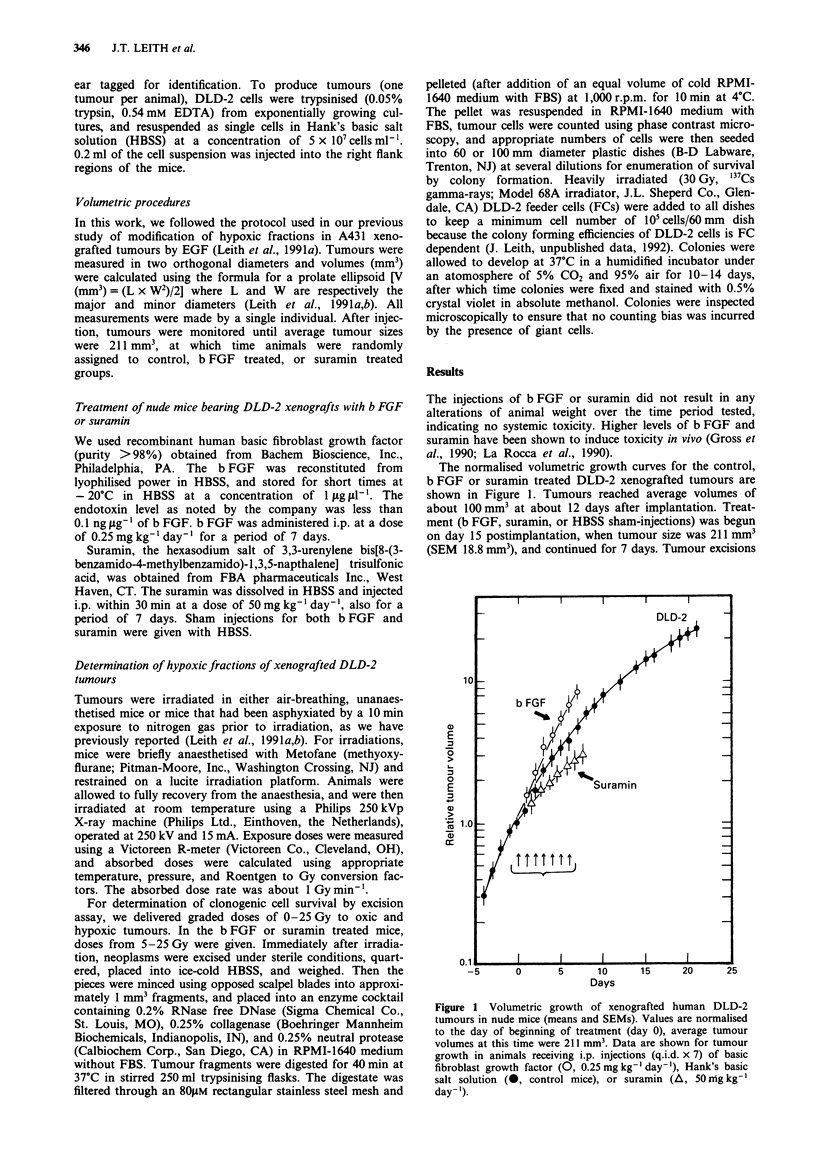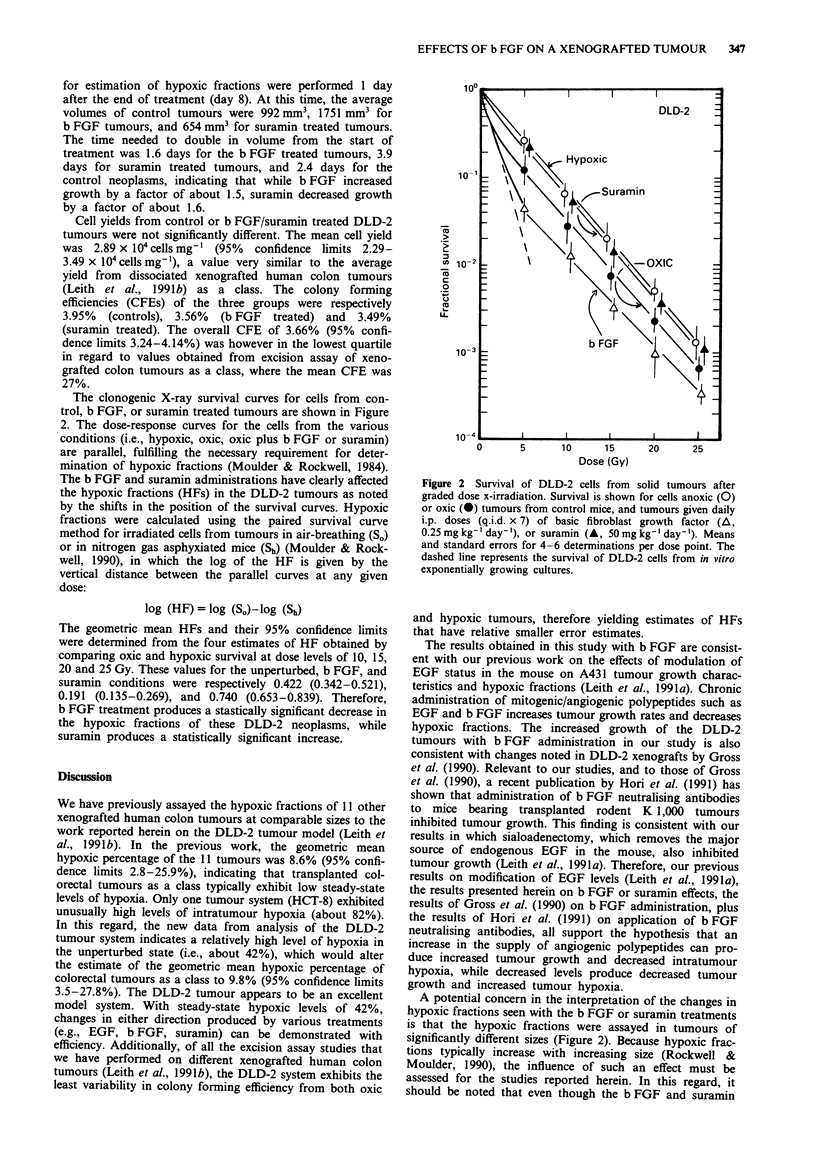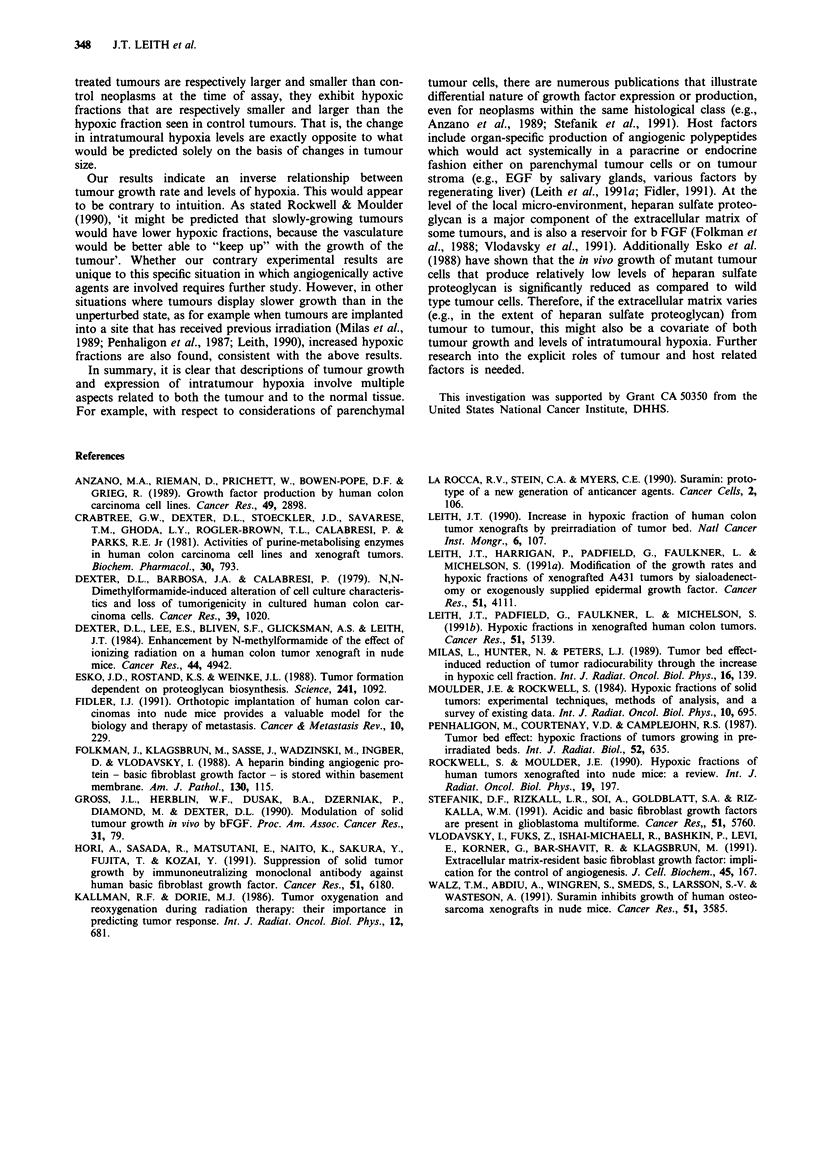# Modification of the volumetric growth responses and steady-state hypoxic fractions of xenografted DLD-2 human colon carcinomas by administration of basic fibroblast growth factor or suramin.

**DOI:** 10.1038/bjc.1992.268

**Published:** 1992-08

**Authors:** J. T. Leith, G. Papa, L. Quaranto, S. Michelson

**Affiliations:** Department of Radiation Medicine, Brown University School of Medicine, Providence, RI 02912.

## Abstract

We studied the growth characteristics and hypoxic fractions of DLD-2 human colon tumours xenografted into male nude mice either in the unperturbed state or after i.p. injection (q.i.d. x 7) of basic fibroblast growth factor (0.25 mg kg-1) or suramin (50 mg kg-1). Hypoxic fractions were measured by clonogenic excision assay 1 day after administration b FGF or suramin was stopped. As compared to controls, the growth of tumours in b FGF treated mice was increased by a factor of 1.5 as indicated by the relative volumes of tumours on the day of excision. Similarly, suramin decreased the growth of DLD-2 tumours by a factor of 1.6. The percentage of hypoxic cells in control neoplasms was 42.9% (95% confidence limits 34.2-52.1%). In mice that received basic fibroblast growth factor injections, hypoxic fractions decreased to 19.1% (95% confidence limits 13.5-26.9%). In contrast, in mice treated with suramin, the percentage of hypoxic cells increased to 74.0% (95% confidence limits 65.3-83.9%). These data indicate that the biology of solid tumours can be significantly modified by alteration of growth factor status.


Br. J. Cancer (1992), 66, 345–348 © Macmillan Press Ltd., 1992

### Modification of the volumetric growth responses and steady-state hypoxic fractions of xenografted DLD-2 human colon carcinomas by administration of basic fibroblast growth factor or suramin

J.T. Leith[1], G. Papa, L. Quaranto & S. Michelson[2]

[1]*Radiation Research Laboratories, Department of Radiation Medicine, Brown University School of Medicine, Providence, RI 02912, USA; and* [2]*Syntex Corporation, Institute for Research Data Management, Palo Alto, CA 94303, USA.*

**Summary** We studied the growth characteristics and hypoxic fractions of DLD-2 human colon tumours xenografted into male nude mice either in the unperturbed state or after i.p. injection (q.i.d. × 7) of basic fibroblast growth factor ($0.25\,mg\,kg^{-1}$) or suramin ($50\,mg\,kg^{-1}$). Hypoxic fractions were measured by clonogenic excision assay 1 day after administration b FGF or suramin was stopped. As compared to controls, the growth of tumours in b FGF treated mice was increased by a factor of 1.5 as indicated by the relative volumes of tumours on the day of excision. Similarly, suramin decreased the growth of DLD-2 tumours by a factor of 1.6. The percentage of hypoxic cells in control neoplasms was 42.9% (95% confidence limits 34.2–52.1%). In mice that received basic fibroblast growth factor injections, hypoxic fractions decreased to 19.1% (95% confidence limits 13.5–26.9%). In contrast, in mice treated with suramin, the percentage of hypoxic cells increased to 74.0% (95% confidence limits 65.3–83.9%). These data indicate that the biology of solid tumours can be significantly modified by alteration of growth factor status.

Moulder and Rockwell (1984) and Rockwell and Moulder (1990) have summarised the extent of hypoxia in various model tumour systems. In these reviews, the authors note that it is important to remember that quoted 'percentages' of hypoxia within tumours represent instantaneous steady-state values within dynamic systems. Examples of this dynamism are the increased hypoxic levels seen as tumour size increases (summarised in Rockwell & Moulder, 1990), and the transients in hypoxic percentages seen during the postirradiation process of reoxygenation (Kallman & Dorie, 1986). In addition, we have recently shown (Leith *et al.*, 1991*a*) that the steady-state levels of hypoxia (about 10.5%) existing within unperturbed xenografted human A431 epidermoid carcinomas could be significantly altered by modification of the host growth factor status. Specifically, removal of the salivary glands in mice, which significantly reduces the circulating levels of epidermal growth factor (EGF), slowed volumetric tumour growth and concomitantly increased hypoxic fractions (to about 35%). Conversely, daily injections of EGF ($0.25\,mg\,kg^{-1}\,day^{-1}$) in nonsialoadenectomised mice increased tumour growth and decreased hypoxic fractions (to about 3.5%). Therefore, systemic levels of growth factors that possess mitogenic and/or angiogenic properties (e.g., EGF) may be important in expression of intraneoplastic hypoxia. As improving tumour response and curability with ionising radiation is a major research focus, manipulation of intratumour hypoxia using growth factors is worthy of further study.

To this end, we have investigated the DLD-2 xenografted human colon tumour system with respect to growth factor modulation of intratumour hypoxia by two additional treatments. First, we administered a different growth factor – basic fibroblast growth factor (b FGF), which has been reported to affect tumour growth (Gross *et al.*, 1990). Second, we administered an agent which blocks growth factor receptors, suramin, and which has been shown to inhibit the growth of xenografted human osteosarcomas (Walz *et al.*, 1991). The reasons for choosing the DLD-2 tumour model for these investigations were 2-fold. First, we have previously studied this tumour system *in vivo*, in regard to unperturbed volumetric growth and radiosensitivity (assayed by tumour growth delay) (Dexter *et al.*, 1984). Second, as stated, Gross *et al.* (1990) have studied volumetric responses of xenografted DLD-2 tumours to daily injection with b FGF and found increased tumour growth rates. Therefore, based on the response of DLD-2 xenografted human colon tumours to b FGF as noted by Gross *et al.* (1990), and on our previous studies on the responses of A431 tumours to FGF, we hypothesised that treatment of DLD-2 tumours with b FGF *in vivo* would decrease hypoxic fractions. Similarly, based on previous results showing decreased hypoxic fractions in A431 tumours after sialoadenectomy, we hypothesised that suramin treatment would increase hypoxic fractions. Our results verify these hypotheses, that is, daily injections of b FGF decreased intratumour hypoxic fractions, and increased volumetric growth rates, while daily injections of suramin decreased growth rates and increased the extent of intratumour hypoxia.

### Materials and methods

### *Cell line*

The DLD-2 human colon adenocarcinoma cell line was established in 1978 at the Roger Williams Cancer Center, Providence, RI from a patient with a well to moderately well differentiated primary adenocarcinoma of the colon. Details on this cell line have been previously published (Crabtree *et al.*, 1981; Dexter *et al.*, 1979). For these experiments, stock cells stored in liquid nitrogen were grown in RPMI-1640 medium containing 10% foetal bovine serum (FBS), 1% sodium bicarbonate, 1% anti-PPLO reagent, 1% 4-(2-hydroxyethyl)-1-piperazineethanesulfonic acid buffer, and 0.04% gentamicin (all reagents from the Grand Island Biological Co., Grand Island, NY).

### *Mice and production of xenografted tumours*

Young adult male nude mice were obtained from the Charles River Breeding Laboratories, North Wilmington, MA. Mice were housed, 5–10 per large cage, with dust covers, in a dedicated room in the Brown University Animal Care Facilities in a laminar flow hood (Thoren Industries, King of Prussia, PA). Mice were quarantined for 1 week, and were

Correspondence: J.T. Leith, Radiation Research Laboratories, Box G, Rm. B-004, Brown University School of Medicine, Providence, RI 02912, USA.
Received 30 January 1992; and in revised form 5 May 1992.

ear tagged for identification. To produce tumours (one tumour per animal), DLD-2 cells were trypsinised (0.05% trypsin, 0.54 mM EDTA) from exponentially growing cultures, and resuspended as single cells in Hank's basic salt solution (HBSS) at a concentration of $5 \times 10^7$ cells $ml^{-1}$. 0.2 ml of the cell suspension was injected into the right flank regions of the mice.

*Volumetric procedures*

In this work, we followed the protocol used in our previous study of modification of hypoxic fractions in A431 xenografted tumours by EGF (Leith *et al.*, 1991*a*). Tumours were measured in two orthogonal diameters and volumes ($mm^3$) were calculated using the formula for a prolate ellipsoid [V ($mm^3$) = $(L \times W^2)/2$] where L and W are respectively the major and minor diameters (Leith *et al.*, 1991*a*,*b*). All measurements were made by a single individual. After injection, tumours were monitored until average tumour sizes were 211 $mm^3$, at which time animals were randomly assigned to control, b FGF treated, or suramin treated groups.

*Treatment of nude mice bearing DLD-2 xenografts with b FGF or suramin*

We used recombinant human basic fibroblast growth factor (purity >98%) obtained from Bachem Bioscience, Inc., Philadelphia, PA. The b FGF was reconstituted from lyophilised power in HBSS, and stored for short times at − 20°C in HBSS at a concentration of 1 $\mu g\, \mu l^{-1}$. The endotoxin level as noted by the company was less than 0.1 ng $\mu g^{-1}$ of b FGF. b FGF was administered i.p. at a dose of 0.25 mg $kg^{-1}$ $day^{-1}$ for a period of 7 days.

Suramin, the hexasodium salt of 3,3-urenylene bis[8-(3-benzamido-4-methylbenzamido)-1,3,5-napthalene] trisulfonic acid, was obtained from FBA pharmaceuticals Inc., West Haven, CT. The suramin was dissolved in HBSS and injected i.p. within 30 min at a dose of 50 mg $kg^{-1}$ $day^{-1}$, also for a period of 7 days. Sham injections for both b FGF and suramin were given with HBSS.

*Determination of hypoxic fractions of xenografted DLD-2 tumours*

Tumours were irradiated in either air-breathing, unanaesthetised mice or mice that had been asphyxiated by a 10 min exposure to nitrogen gas prior to irradiation, as we have previously reported (Leith *et al.*, 1991*a*,*b*). For irradiations, mice were briefly anaesthetised with Metofane (methyoxyflurane; Pitman-Moore, Inc., Washington Crossing, NJ) and restrained on a lucite irradiation platform. Animals were allowed to fully recovery from the anaesthesia, and were then irradiated at room temperature using a Philips 250 kVp X-ray machine (Philips Ltd., Einthoven, the Netherlands), operated at 250 kV and 15 mA. Exposure doses were measured using a Victoreen R-meter (Victoreen Co., Cleveland, OH), and absorbed doses were calculated using appropriate temperature, pressure, and Roentgen to Gy conversion factors. The absorbed dose rate was about 1 Gy $min^{-1}$.

For determination of clonogenic cell survival by excision assay, we delivered graded doses of 0–25 Gy to oxic and hypoxic tumours. In the b FGF or suramin treated mice, doses from 5–25 Gy were given. Immediately after irradiation, neoplasms were excised under sterile conditions, quartered, placed into ice-cold HBSS, and weighed. Then the pieces were minced using opposed scalpel blades into approximately 1 $mm^3$ fragments, and placed into an enzyme cocktail containing 0.2% RNase free DNase (Sigma Chemical Co., St. Louis, MO), 0.25% collagenase (Boehringer Mannheim Biochemicals, Indianopolis, IN), and 0.25% neutral protease (Calbiochem Corp., San Diego, CA) in RPMI-1640 medium without FBS. Tumour fragments were digested for 40 min at 37°C in stirred 250 ml trypsinising flasks. The digestate was filtered through an 80μM rectangular stainless steel mesh and pelleted (after addition of an equal volume of cold RPMI-1640 medium with FBS) at 1,000 r.p.m. for 10 min at 4°C. The pellet was resuspended in RPMI-1640 medium with FBS, tumour cells were counted using phase contrast microscopy, and appropriate numbers of cells were then seeded into 60 or 100 mm diameter plastic dishes (B-D Labware, Trenton, NJ) at several dilutions for enumeration of survival by colony formation. Heavily irradiated (30 Gy, $^{137}Cs$ gamma-rays; Model 68A irradiator, J.L. Sheperd Co., Glendale, CA) DLD-2 feeder cells (FCs) were added to all dishes to keep a minimum cell number of $10^5$ cells/60 mm dish because the colony forming efficiencies of DLD-2 cells is FC dependent (J. Leith, unpublished data, 1992). Colonies were allowed to develop at 37°C in a humidified incubator under an atomosphere of 5% $CO_2$ and 95% air for 10–14 days, after which time colonies were fixed and stained with 0.5% crystal violet in absolute methanol. Colonies were inspected microscopically to ensure that no counting bias was incurred by the presence of giant cells.

### Results

The injections of b FGF or suramin did not result in any alterations of animal weight over the time period tested, indicating no systemic toxicity. Higher levels of b FGF and suramin have been shown to induce toxicity *in vivo* (Gross *et al.*, 1990; La Rocca *et al.*, 1990).

The normalised volumetric growth curves for the control, b FGF or suramin treated DLD-2 xenografted tumours are shown in Figure 1. Tumours reached average volumes of about 100 $mm^3$ at about 12 days after implantation. Treatment (b FGF, suramin, or HBSS sham-injections) was begun on day 15 postimplantation, when tumour size was 211 $mm^3$ (SEM 18.8 $mm^3$), and continued for 7 days. Tumour excisions

**Figure 1** Volumetric growth of xenografted human DLD-2 tumours in nude mice (means and SEMs). Values are normalised to the day of beginning of treatment (day 0), average tumour volumes at this time were 211 $mm^3$. Data are shown for tumour growth in animals receiving i.p. injections (q.i.d. × 7) of basic fibroblast growth factor (O, 0.25 mg $kg^{-1}$ $day^{-1}$), Hank's basic salt solution (●, control mice), or suramin (Δ, 50 mg $kg^{-1}$ $day^{-1}$).

for estimation of hypoxic fractions were performed 1 day after the end of treatment (day 8). At this time, the average volumes of control tumours were 992 $mm^3$, 1751 $mm^3$ for b FGF tumours, and 654 $mm^3$ for suramin treated tumours. The time needed to double in volume from the start of treatment was 1.6 days for the b FGF treated tumours, 3.9 days for suramin treated tumours, and 2.4 days for the control neoplasms, indicating that while b FGF increased growth by a factor of about 1.5, suramin decreased growth by a factor of about 1.6.

Cell yields from control or b FGF/suramin treated DLD-2 tumours were not significantly different. The mean cell yield was $2.89 \times 10^4$ cells $mg^{-1}$ (95% confidence limits 2.29–3.49 $\times 10^4$ cells $mg^{-1}$), a value very similar to the average yield from dissociated xenografted human colon tumours (Leith *et al.*, 1991*b*) as a class. The colony forming efficiencies (CFEs) of the three groups were respectively 3.95% (controls), 3.56% (b FGF treated) and 3.49% (suramin treated). The overall CFE of 3.66% (95% confidence limits 3.24–4.14%) was however in the lowest quartile in regard to values obtained from excision assay of xenografted colon tumours as a class, where the mean CFE was 27%.

The clonogenic X-ray survival curves for cells from control, b FGF, or suramin treated tumours are shown in Figure 2. The dose-response curves for the cells from the various conditions (i.e., hypoxic, oxic, oxic plus b FGF or suramin) are parallel, fulfilling the necessary requirement for determination of hypoxic fractions (Moulder & Rockwell, 1984). The b FGF and suramin administrations have clearly affected the hypoxic fractions (HFs) in the DLD-2 tumours as noted by the shifts in the position of the survival curves. Hypoxic fractions were calculated using the paired survival curve method for irradiated cells from tumours in air-breathing ($S_o$) or in nitrogen gas asphyxiated mice ($S_h$) (Moulder & Rockwell, 1990), in which the log of the HF is given by the vertical distance between the parallel curves at any given dose:

$$\log(\text{HF}) = \log(S_o) - \log(S_h)$$

The geometric mean HFs and their 95% confidence limits were determined from the four estimates of HF obtained by comparing oxic and hypoxic survival at dose levels of 10, 15, 20 and 25 Gy. These values for the unperturbed, b FGF, and suramin conditions were respectively 0.422 (0.342–0.521), 0.191 (0.135–0.269), and 0.740 (0.653–0.839). Therefore, b FGF treatment produces a stastically significant decrease in the hypoxic fractions of these DLD-2 neoplasms, while suramin produces a statistically significant increase.

### Discussion

We have previously assayed the hypoxic fractions of 11 other xenografted human colon tumours at comparable sizes to the work reported herein on the DLD-2 tumour model (Leith *et al.*, 1991*b*). In the previous work, the geometric mean hypoxic percentage of the 11 tumours was 8.6% (95% confidence limits 2.8–25.9%), indicating that transplanted colorectal tumours as a class typically exhibit low steady-state levels of hypoxia. Only one tumour system (HCT-8) exhibited unusually high levels of intratumour hypoxia (about 82%). In this regard, the new data from analysis of the DLD-2 tumour system indicates a relatively high level of hypoxia in the unperturbed state (i.e., about 42%), which would alter the estimate of the geometric mean hypoxic percentage of colorectal tumours as a class to 9.8% (95% confidence limits 3.5–27.8%). The DLD-2 tumour appears to be an excellent model system. With steady-state hypoxic levels of 42%, changes in either direction produced by various treatments (e.g., EGF, b FGF, suramin) can be demonstrated with efficiency. Additionally, of all the excision assay studies that we have performed on different xenografted human colon tumours (Leith *et al.*, 1991*b*), the DLD-2 system exhibits the least variability in colony forming efficiency from both oxic

**Figure 2** Survival of DLD-2 cells from solid tumours after graded dose x-irradiation. Survival is shown for cells anoxic (○) or oxic (●) tumours from control mice, and tumours given daily i.p. doses (q.i.d. × 7) of basic fibroblast growth factor (Δ, 0.25 mg $kg^{-1}$ $day^{-1}$), or suramin (▲, 50 mg $kg^{-1}$ $day^{-1}$). Means and standard errors for 4–6 determinations per dose point. The dashed line represents the survival of DLD-2 cells from *in vitro* exponentially growing cultures.

and hypoxic tumours, therefore yielding estimates of HFs that have relative smaller error estimates.

The results obtained in this study with b FGF are consistent with our previous work on the effects of modulation of EGF status in the mouse on A431 tumour growth characteristics and hypoxic fractions (Leith *et al.*, 1991*a*). Chronic administration of mitogenic/angiogenic polypeptides such as EGF and b FGF increases tumour growth rates and decreases hypoxic fractions. The increased growth of the DLD-2 tumours with b FGF administration in our study is also consistent with changes noted in DLD-2 xenografts by Gross *et al.* (1990). Relevant to our studies, and to those of Gross *et al.* (1990), a recent publication by Hori *et al.* (1991) has shown that administration of b FGF neutralising antibodies to mice bearing transplanted rodent K 1,000 tumours inhibited tumour growth. This finding is consistent with our results in which sialoadenectomy, which removes the major source of endogenous EGF in the mouse, also inhibited tumour growth (Leith *et al.*, 1991*a*). Therefore, our previous results on modification of EGF levels (Leith *et al.*, 1991*a*), the results presented herein on b FGF or suramin effects, the results of Gross *et al.* (1990) on b FGF administration, plus the results of Hori *et al.* (1991) on application of b FGF neutralising antibodies, all support the hypothesis that an increase in the supply of angiogenic polypeptides can produce increased tumour growth and decreased intratumour hypoxia, while decreased levels produce decreased tumour growth and increased tumour hypoxia.

A potential concern in the interpretation of the changes in hypoxic fractions seen with the b FGF or suramin treatments is that the hypoxic fractions were assayed in tumours of significantly different sizes (Figure 2). Because hypoxic fractions typically increase with increasing size (Rockwell & Moulder, 1990), the influence of such an effect must be assessed for the studies reported herein. In this regard, it should be noted that even though the b FGF and suramin treated tumours are respectively larger and smaller than control neoplasms at the time of assay, they exhibit hypoxic fractions that are respectively smaller and larger than the hypoxic fraction seen in control tumours. That is, the change in intratumoural hypoxia levels are exactly opposite to what would be predicted solely on the basis of changes in tumour size.

Our results indicate an inverse relationship between tumour growth rate and levels of hypoxia. This would appear to be contrary to intuition. As stated Rockwell & Moulder (1990), 'it might be predicted that slowly-growing tumours would have lower hypoxic fractions, because the vasculature would be better able to "keep up" with the growth of the tumour'. Whether our contrary experimental results are unique to this specific situation in which angiogenically active agents are involved requires further study. However, in other situations where tumours display slower growth than in the unperturbed state, as for example when tumours are implanted into a site that has received previous irradiation (Milas et al., 1989; Penhaligon et al., 1987; Leith, 1990), increased hypoxic fractions are also found, consistent with the above results.

In summary, it is clear that descriptions of tumour growth and expression of intratumour hypoxia involve multiple aspects related to both the tumour and to the normal tissue. For example, with respect to considerations of parenchymal tumour cells, there are numerous publications that illustrate differential nature of growth factor expression or production, even for neoplasms within the same histological class (e.g., Anzano et al., 1989; Stefanik et al., 1991). Host factors include organ-specific production of angiogenic polypeptides which would act systemically in a paracrine or endocrine fashion either on parenchymal tumour cells or on tumour stroma (e.g., EGF by salivary glands, various factors by regenerating liver) (Leith et al., 1991a; Fidler, 1991). At the level of the local micro-environment, heparan sulfate proteoglycan is a major component of the extracellular matrix of some tumours, and is also a reservoir for b FGF (Folkman et al., 1988; Vlodavsky et al., 1991). Additionally Esko et al. (1988) have shown that the *in vivo* growth of mutant tumour cells that produce relatively low levels of heparan sulfate proteoglycan is significantly reduced as compared to wild type tumour cells. Therefore, if the extracellular matrix varies (e.g., in the extent of heparan sulfate proteoglycan) from tumour to tumour, this might also be a covariate of both tumour growth and levels of intratumoural hypoxia. Further research into the explicit roles of tumour and host related factors is needed.

This investigation was supported by Grant CA 50350 from the United States National Cancer Institute, DHHS.

### References

ANZANO, M.A., RIEMAN, D., PRICHETT, W., BOWEN-POPE, D.F. & GRIEG, R. (1989). Growth factor production by human colon carcinoma cell lines. *Cancer Res.*, **49**, 2898.

CRABTREE, G.W., DEXTER, D.L., STOECKLER, J.D., SAVARESE, T.M., GHODA, L.Y., ROGLER-BROWN, T.L., CALABRESI, P. & PARKS, R.E. Jr (1981). Activities of purine-metabolising enzymes in human colon carcinoma cell lines and xenograft tumors. *Biochem. Pharmacol.*, **30**, 793.

DEXTER, D.L., BARBOSA, J.A. & CALABRESI, P. (1979). N,N-Dimethylformamide-induced alteration of cell culture characteristics and loss of tumorigenicity in cultured human colon carcinoma cells. *Cancer Res.*, **39**, 1020.

DEXTER, D.L., LEE, E.S., BLIVEN, S.F., GLICKSMAN, A.S. & LEITH, J.T. (1984). Enhancement by N-methylformamide of the effect of ionizing radiation on a human colon tumor xenograft in nude mice. *Cancer Res.*, **44**, 4942.

ESKO, J.D., ROSTAND, K.S. & WEINKE, J.L. (1988). Tumor formation dependent on proteoglycan biosynthesis. *Science*, **241**, 1092.

FIDLER, I.J. (1991). Orthotopic implantation of human colon carcinomas into nude mice provides a valuable model for the biology and therapy of metastasis. *Cancer & Metastasis Rev.*, **10**, 229.

FOLKMAN, J., KLAGSBRUN, M., SASSE, J., WADZINSKI, M., INGBER, D. & VLODAVSKY, I. (1988). A heparin binding angiogenic protein – basic fibroblast growth factor – is stored within basement membrane. *Am. J. Pathol.*, **130**, 115.

GROSS, J.L., HERBLIN, W.F., DUSAK, B.A., DZERNIAK, P., DIAMOND, M. & DEXTER, D.L. (1990). Modulation of solid tumour growth *in vivo* by bFGF. *Proc. Am. Assoc. Cancer Res.*, **31**, 79.

HORI, A., SASADA, R., MATSUTANI, E., NAITO, K., SAKURA, Y., FUJITA, T. & KOZAI, Y. (1991). Suppression of solid tumor growth by immunoneutralizing monoclonal antibody against human basic fibroblast growth factor. *Cancer Res.*, **51**, 6180.

KALLMAN, R.F. & DORIE, M.J. (1986). Tumor oxygenation and reoxygenation during radiation therapy: their importance in predicting tumor response. *Int. J. Radiat. Oncol. Biol. Phys.*, **12**, 681.

LA ROCCA, R.V., STEIN, C.A. & MYERS, C.E. (1990). Suramin: prototype of a new generation of anticancer agents. *Cancer Cells*, **2**, 106.

LEITH, J.T. (1990). Increase in hypoxic fraction of human colon tumor xenografts by preirradiation of tumor bed. *Natl Cancer Inst. Mongr.*, **6**, 107.

LEITH, J.T., HARRIGAN, P., PADFIELD, G., FAULKNER, L. & MICHELSON, S. (1991*a*). Modification of the growth rates and hypoxic fractions of xenografted A431 tumors by sialoadenectomy or exogenously supplied epidermal growth factor. *Cancer Res.*, **51**, 4111.

LEITH, J.T., PADFIELD, G., FAULKNER, L. & MICHELSON, S. (1991*b*). Hypoxic fractions in xenografted human colon tumors. *Cancer Res.*, **51**, 5139.

MILAS, L., HUNTER, N. & PETERS, L.J. (1989). Tumor bed effect-induced reduction of tumor radiocurability through the increase in hypoxic cell fraction. *Int. J. Radiat. Oncol. Biol. Phys.*, **16**, 139.

MOULDER, J.E. & ROCKWELL, S. (1984). Hypoxic fractions of solid tumors: experimental techniques, methods of analysis, and a survey of existing data. *Int. J. Radiat. Oncol. Biol. Phys.*, **10**, 695.

PENHALIGON, M., COURTENAY, V.D. & CAMPLEJOHN, R.S. (1987). Tumor bed effect: hypoxic fractions of tumors growing in pre-irradiated beds. *Int. J. Radiat. Biol.*, **52**, 635.

ROCKWELL, S. & MOULDER, J.E. (1990). Hypoxic fractions of human tumors xenografted into nude mice: a review. *Int. J. Radiat. Oncol. Biol. Phys.*, **19**, 197.

STEFANIK, D.F., RIZKALL, L.R., SOI, A., GOLDBLATT, S.A. & RIZKALLA, W.M. (1991). Acidic and basic fibroblast growth factors are present in glioblastoma multiforme. *Cancer Res,*, **51**, 5760.

VLODAVSKY, I., FUKS, Z., ISHAI-MICHAELI, R., BASHKIN, P., LEVI, E., KORNER, G., BAR-SHAVIT, R. & KLAGSBRUN, M. (1991). Extracellular matrix-resident basic fibroblast growth factor: implication for the control of angiogenesis. *J. Cell. Biochem.*, **45**, 167.

WALZ, T.M., ABDIU, A., WINGREN, S., SMEDS, S., LARSSON, S.-V. & WASTESON, A. (1991). Suramin inhibits growth of human osteosarcoma xenografts in nude mice. *Cancer Res.*, **51**, 3585.